# Improving the Understandability of Clinical Guidelines: Development and Evaluation of a GPT-4–Based Pipeline

**DOI:** 10.2196/81915

**Published:** 2026-02-23

**Authors:** Matthew D Jones, Melissa Torgbi, Harish Tayyar Madabushi

**Affiliations:** 1 Department of Life Sciences, University of Bath, Claverton Down, Bath, BA2 7AY, United Kingdom, 44 1225383829; 2 Department of Computer Science, University of Bath, Bath, United Kingdom

**Keywords:** artificial intelligence, clinical guidelines, comprehension, guidelines as topic, health care professionals, health personnel, large language models, patient safety, readability, understandability

## Abstract

**Background:**

Difficulty in finding and understanding information in clinical guidelines contributes to medication errors. Large language models (LLMs) can simplify complex text to aid in understanding, but this approach to improving the quality of guidelines has not been investigated. However, LLMs are also known to hallucinate or generate outputs that may not align with reality.

**Objective:**

This study aimed to develop and evaluate an LLM pipeline to improve the readability of clinical guidelines while ensuring the preservation of critical content.

**Methods:**

To align LLM revisions with research evidence and enable comparison with manual editing, the National Health Service Injectable Medicines Guide (IMG) was used as a case study, to which a GPT-4–based pipeline was applied, with prompts based on user testing–derived recommendations for IMG authors. This enabled readability comparisons between various IMG guideline versions: original, manually revised, or GPT-4–revised using the user testing–derived recommendations, and fully user tested. Readability was evaluated using readability metrics and ratings from 3 expert pharmacists. Content similarity before and after LLM revision was assessed using BERT (bidirectional encoder representations from transformers) scores and expert pharmacist review.

**Results:**

Considering 20 IMG guidelines used in practice, BERT scores indicated high semantic similarity between the original and LLM-revised guidelines (0.88-0.96). An omission, addition, or change in meaning was identified by at least one pharmacist in 30 (20%), 7 (5%), and 18 (12%) of the 153 guideline subsections, respectively. The SMOG (Simple Measure of Gobbledygook) grade showed a small but significant improvement in readability for the LLM-revised guidelines (mean difference 0.32, 95% CI 0.10‐0.55; *P*=.02) and the manually revised versions (mean difference 0.46, 95% CI 0.13‐0.79; *P*=.03). There was no significant difference between the LLM and manually revised versions (*P*>.99). There were no significant differences between Flesch-Kincaid reading grades (*P*=.91). Expert ratings favored the LLM-revised versions for understandability. Considering 2 IMG guidelines from previous research, user testing produced a greater improvement in readability than LLM revision.

**Conclusions:**

Authors should not use current LLMs to modify clinical guidelines without carefully checking the revised text for unintended omissions, additions, or changes in meaning. Further work should investigate the potential of LLMs to augment manual user testing and reduce the barriers to the wider use of this approach to improve the safety of clinical guidelines.

## Introduction

Medication errors are a leading cause of avoidable harm in health care systems, with an estimated international cost of US $42 billion per annum [1]. Numerous factors can lead to a medication error [2], one of which is difficulty in finding or understanding prescribing and medication administration information in clinical guidelines [3-5]. Consequently, guidelines that are contradictory, incomprehensible, or of poor quality are included in frameworks of the causes of patient safety incidents [6]. Clarity of writing and formatting also influences guideline adoption [7].

Few studies have explored ways to help clinicians more easily find and understand guidelines. Some evidence suggests that iterative user testing and redesign can improve guideline usability by identifying and addressing problem areas [8,9]. In one study, user-tested injectable medicines guidelines led to a 2.5-fold increase in error-free intravenous administration and a 96% probability of cost-effectiveness [10,11]. Such improvements result from both the testing process and the application of good practice in information writing and design [12]. Consequently, in 2020, format changes and clear writing advice were added to the authors’ instructions for the United Kingdom’s National Health Service Injectable Medicines Guide (IMG). This is an online guide to preparing and administering injectable medicines, mainly used by nurses. Subsequently, more than 300 drug guidelines have been manually updated, but not user tested.

Most guideline-related medication errors involve documents produced by local health care organizations [3]. User testing and revising these “local guidelines” could therefore yield the greatest safety improvements. As there are many thousands of these documents within a single country, limited expertise in information writing and design within health care organizations may hinder this approach.

Large language models (LLMs), such as GPT-4, may help overcome this barrier by simplifying complex text. This approach has been tested on health information for patients initially written by subject experts. Early results are mixed: LLMs can improve readability metrics [13-18] and patient understanding [15] but can also worsen readability metrics [19]. These gains may come at a cost, with some content omitted and up to 21% of revised content deemed clinically inappropriate [13-15,19]. LLMs have also been used to improve understanding of specialist medical records [20]. However, they have not yet been applied to improve the readability of clinical guidelines for health care professionals; therefore, it remains unclear whether LLMs can enhance readability without compromising content accuracy or safety.

**Figure 1.**
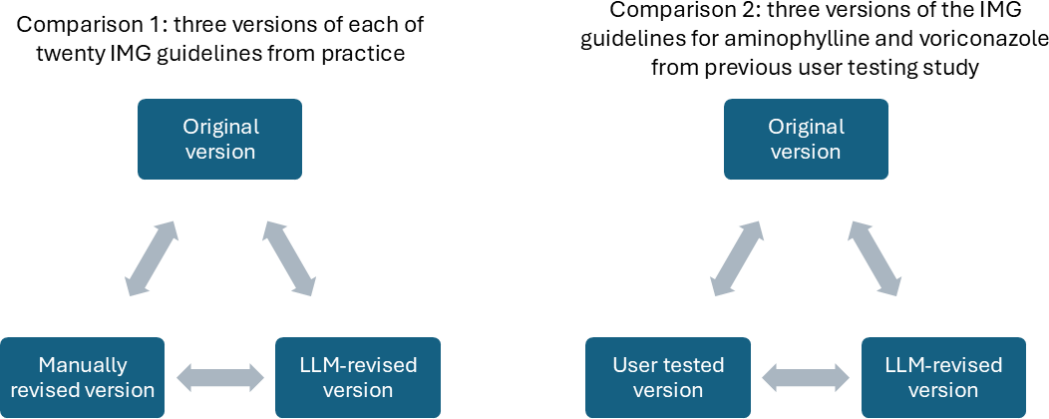
Readability comparisons reported in this study. “Original” versions were the guidelines in use in the Injectable Medicines Guide (IMG) in 2019. “LLM-revised” versions were developed from the original guidelines by the large language model (LLM) pipeline. “Manually revised” versions were the guidelines in use in the IMG in 2024, which had been updated by guideline authors in line with revised authors’ instructions introduced in 2020. “User-tested” versions were developed from the original guidelines via a user-testing process during a previous study [9].

Therefore, this study evaluated a GPT-4–based pipeline to improve guideline readability while safeguarding against omission, addition, or change of meaning of safety-critical content. To enable comparison with manual revision and align with previous research, the pipeline was applied to the IMG. This enabled various readability comparisons (Figure 1), as well as comparison of the content of guidelines before and after LLM revision.

Specific objectives were therefore to (1) develop a GPT-4–based pipeline to improve guideline clarity with built-in safeguards; (2) evaluate content similarity of 20 guidelines before and after revision by the GPT-4 pipeline; (3) compare the readability of 20 equivalent original guidelines, LLM-revised guidelines, and manually revised guidelines; and (4) compare the readability of original, LLM-revised, and user-tested aminophylline and voriconazole guidelines.

## Methods

This study is reported in accordance with the TRIPOD-LLM (Transparent Reporting of a Multivariable Model for Individual Prognosis or Diagnosis - Large Language Model) guideline [21].

### Development and Application of the GPT-4 Pipeline

IMG guidelines were converted to Markdown and paired with iteratively developed prompts for input to the LLM pipeline. Prompts instructed the LLM to improve clarity and readability without altering content. The LLM pipeline used the OpenAI API and the GPT-4 model. GPT-4–generated “LLM-revised” Markdown versions of the original guidelines, which were then converted to static HTML for display.

### Prompting Strategies

Prompting was based on the Reframing Principles for Instruction-Following LLMs [22], which offer tested strategies for constructing effective prompts, including using low-level patterns, examples, simplified sentence structures, and clear constraints. Initially, researchers with expertise in computer science (MT and HTM) constructed prompts based on the user testing–derived recommendations incorporated into the IMG authors’ instructions in 2020 [9]. To prevent omissions or changes in meaning, prompts required a semantic similarity score above 0.9 before proceeding. While GPT-4 cannot compute semantic accuracy, we hypothesized that it would interpret this instruction to ensure edits were semantically close to the original and that this reference would make the requirement more explicit. Selected outputs of these initial prompts were reviewed by a pharmacist with expertise in intravenous medication administration and information design (MDJ) and discussed with the wider research team. These initial tests found that overly prescriptive instructions for human authors caused overgeneralization and errors, including omissions and additions. Prompt instructions were then iteratively refined to improve effectiveness. The final prompts (Multimedia Appendix 1, online supplement) used for both comparisons were a product of this iterative development, incorporating insights from our explorations while omitting some instructions originally intended for human authors.

GPT-4–generated instructions were also explored for comparison 2 (Figure 1). The original and user-tested guidelines for voriconazole and aminophylline were given to GPT-4, which was instructed to generate prompts for another model to revise the original guidelines to be similar to the user-tested versions, without directly referencing them. However, these prompts were ineffective and were therefore excluded from final experiments.

### Selection of IMG Guidelines

Twenty adult IMG guidelines were selected in consultation with the IMG editorial team to cover a range of drugs, administration methods (infusion and injection), reconstitution or dilution needs, and higher-risk or complex cases. Versions from 2019 (“original”) and 2024 (“manually revised,” updated in line with revised IMG authors’ instructions) were obtained. Voriconazole and aminophylline guidelines from 2019 and their user-tested revisions were also sourced from prior research [9].

### Guideline Content Similarity

Content similarity of the 20 IMG guidelines from practice before and after LLM revision was assessed using the bidirectional encoder representations from transformers (BERT) score [23] and expert review. The BERT score calculates the similarity between each word or token in a candidate and reference sentence using cosine similarity and the contextual embeddings from the BERT model [20]. We used the standard BERT score library to perform this calculation [24]. Scores range from 0 to 1, with higher values indicating greater similarity.

Three intensive care pharmacists independently assessed 9 sections of LLM-revised guidelines (method of administration, reconstitution, dilution, expiry, flushing, adverse effects, extravasation, other comments, and compatibility) for clinically relevant omissions, additions, or changes in meaning compared with the originals. The pharmacists were known to the research team through professional networks and selected for their significant experience in intensive care pharmacy, although this was not formally defined. All invited pharmacists participated and were aware of the study aims, although they were blinded to guideline version while assessing content.

In addition, the outputs were evaluated manually by the research team for modifications to medication names or their order of presentation.

### Guideline Readability

Readability was assessed with 2 metrics and expert review. The readability metrics used were the SMOG (Simple Measure of Gobbledygook) grade and the Flesch-Kincaid reading grade. SMOG was chosen for its validation against full comprehension, while Flesch-Kincaid is widely used in health research [25]. Both estimate the years of education required to understand a text, so larger scores indicate lower readability. Scores were calculated with the Textstat Python library using the entire guideline text.

The relative readability of the 20 IMG guidelines from practice before and after LLM revision was also independently assessed by the 3 intensive care pharmacists. Comparing the original and revised versions of each guideline, they rated relative overall and subsection-level understandability using a 5-point Likert scale.

### Statistical Analysis

Analyses were performed in Stata v18.0 (StataCorp LLC). Readability metrics and BERT scores were summarized as mean (SD), with differences tested using repeated measures ANOVA with post-hoc Bonferroni-corrected paired *t* tests. Expert ratings were summarized descriptively, and interrater reliability assessed using Gwet’s AC to account for skewed distributions [26].

### Ethical Considerations

This study was assessed as having low potential to do harm and thus reviewed and given a favorable opinion by proportionate review by the Department of Life Science Departmental Research Ethics Officer at the University of Bath on June 22, 2023 (number 442).

## Results

### Pipeline Development

The LLM pipeline successfully produced output text showing revised versions of the 20 IMG guidelines from practice and the voriconazole and aminophylline guidelines from previous user-testing research. These revised guidelines were then evaluated for content similarity using the methods described earlier.

### Content Similarity of the Set of 20 Guidelines From Practice

**Figure 2.**
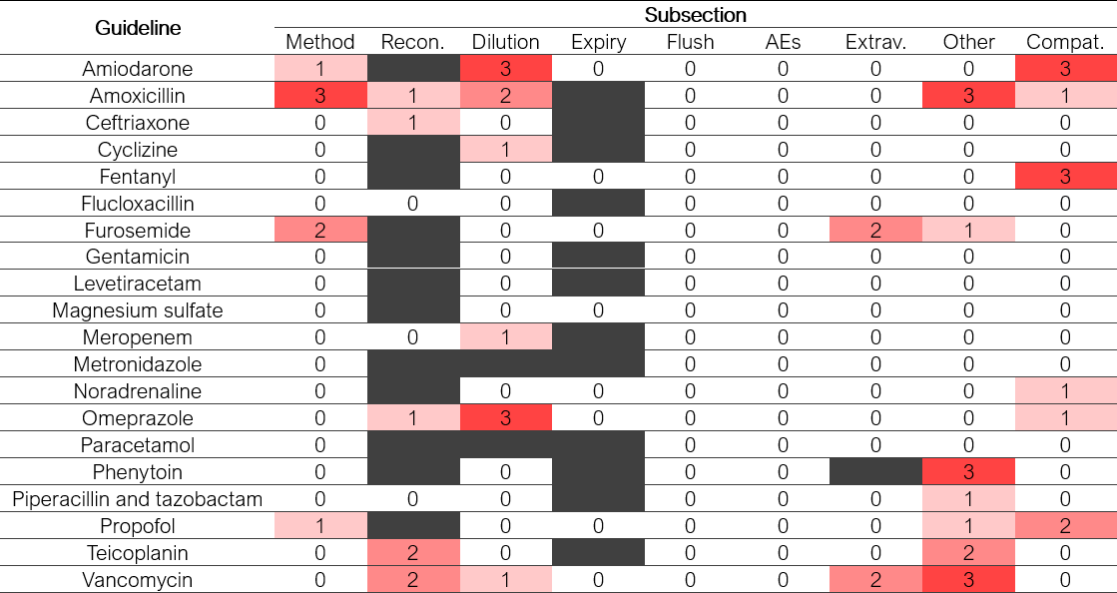
Heatmap showing the number of pharmacist reviewers who identified an omission of information following large language model revision of each subsection of each of the 20 original guidelines. Blank subsections were not present in the relevant guideline. AE: adverse effects; Compat.: compatibility; Expiry: expiry time; Extrav.: extravasation; Flush: flushing; Method: method of administration; Other: other comments; Recon.: reconstitution.

Table 1 presents the BERT scores comparing the semantic similarity of the 20 IMG guidelines from practice before and after LLM revision. These ranged from 0.88 to 0.96, indicating a high degree of similarity. As a benchmark, Hanna and Bojar [27] reported an average BERT score of 0.815 when comparing 2 different, professional human translations of the same source sentences across several distinct linguistic phenomena. The 3 pharmacists’ ratings of clinically relevant omissions, additions, or changes in meaning following LLM revision of the 20 guidelines from practice showed a high degree of interrater reliability, with Gwet’s ACs of 0.87 (omissions), 0.97 (additions), and 0.92 (changes in meaning). Figures2^4^ show the number of pharmacist reviewers who identified an omission, addition, or change in meaning in each subsection. Of 459 individual subsection ratings (153 subsections each rated by 3 pharmacists), 54 (11.8%) identified an omission, 8 (1.7%) identified an addition, and 20 (4.4%) identified a change in meaning. An omission, addition, or change in meaning was identified by at least one pharmacist in 30 (20%), 7 (5%), and 18 (12%), respectively, of the 153 individual subsections. However, only 8 subsections had an omission identified by all 3 reviewers, and no additions or changes of meaning were identified by all 3 reviewers (Table 2). Overall, 65% (36/55) of subsection omissions, additions, or changes of meaning were only identified by 1 reviewer. These differences were concentrated in certain subsections (method of administration, reconstitution, dilution, adverse effects, and other comments) and guidelines (amiodarone, amoxicillin, furosemide, and propofol). Only one subsection (flushing) and two guidelines (levetiracetam and paracetamol) had no omissions, additions, or changes in meaning identified by any pharmacist.

**Table 1.** BERT (bidirectional encoder representations from transformers) scores comparing the semantic similarity of the 20 Injectable Medicines Guide guidelines from practice before and after large language model revision.^a^

Guideline	BERT score
Amiodarone	0.95
Amoxicillin	0.91
Ceftriaxone	0.95
Cyclizine	0.91
Fentanyl	0.96
Flucloxacillin	0.90
Furosemide	0.88
Gentamicin	0.93
Levetiracetam	0.92
Magnesium sulfate	0.90
Meropenem	0.93
Metronidazole	0.90
Noradrenaline	0.94
Omeprazole	0.94
Paracetamol	0.94
Phenytoin	0.88
Piperacillin and tazobactam	0.95
Propofol	0.91
Teicoplanin	0.93
Vancomycin	0.88

a Mean (SD): 0.92 (0.02).

**Figure 3.**
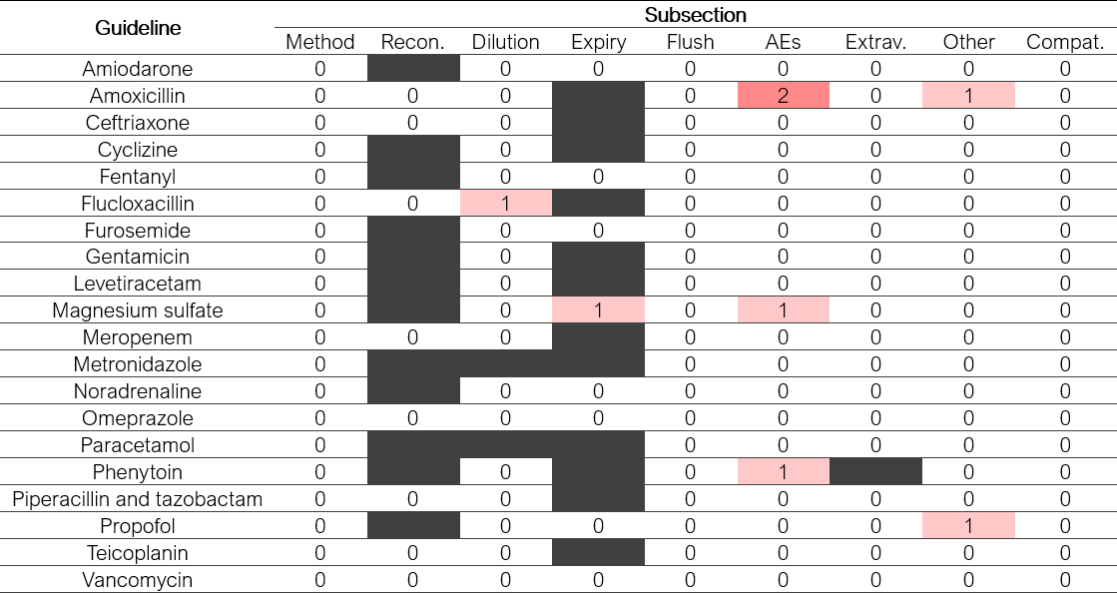
Heatmap showing the number of pharmacist reviewers who identified an addition of information following large language model revision of each subsection of each of the 20 original guidelines. Blank subsections were not present in the relevant guideline. AE: adverse effects; Compat.: compatibility; Expiry: expiry time; Extrav.: extravasation; Flush: flushing; Method: method of administration; Other: other comments; Recon.: reconstitution.

**Figure 4.**
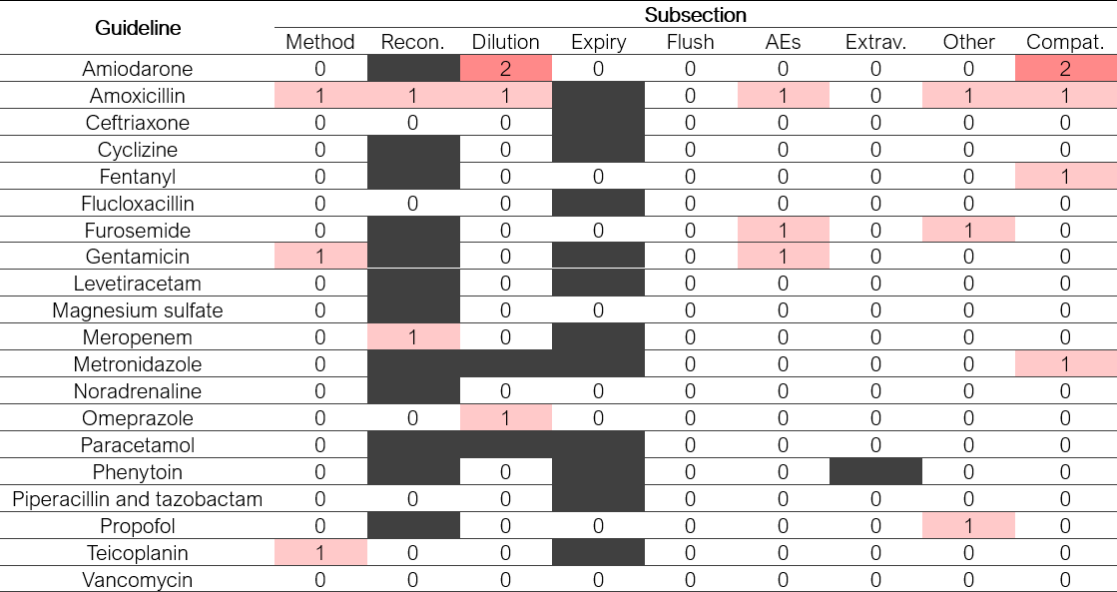
Heatmap showing the number of pharmacist reviewers who identified a change in meaning following large language model revision of each subsection of each of the 20 original guidelines. Blank subsections were not present in the relevant guideline. AEs: adverse effects; Compat.: compatibility; Expiry: expiry time; Extrav.: extravasation; Flush: flushing; Method: method of administration; Other: other comments; Recon.: reconstitution.

**Table 2.** The number of subsections with an identified omission, addition, or change of meaning following large language model revision of each of the 20 original guidelines.

	The number of subsections with an addition, omission, or change of meaning		
	Identified by 1 reviewer	Identified by 2 reviewers	Identified by 3 reviewers
Omission	14	8	8
Addition	6	1	0
Change	16	2	0

Key differences described in the pharmacists’ free text comments included the occasional omission of the contents of an entire subsection (eg, extravasation, other comments, and compatibility), the omission of one specific detail (eg, information relating to one of several doses of a medicine), objective changes in meaning (eg, “hypotension” changed to “hypertension” and “mg/mL” changed to “mg”) and subjective changes in interpretation (eg, the revised wording implying an adverse effect is probable rather than possible or lessening a safety warning).

Manual checks identified no alterations to medication names or order of presentation.

### Readability of the 20 Guidelines From Practice

Table 3 presents overall readability metrics for the 3 different versions of the IMG from practice. Table S1 in Multimedia Appendix 1 shows the metrics calculated for each individual guideline. Repeated measures ANOVA identified a significant difference between the SMOG grades of at least 2 guideline versions (*P*=.007; df=59), so post hoc paired *t* tests with Bonferroni corrections were performed. The mean difference in SMOG grade between the original and LLM-revised versions was 0.32 (95% CI 0.10‐0.55; *P*=.02; df=19), indicating a small but significant improvement in readability following LLM revision. Similarly, the mean difference in SMOG grade between the original and manually revised versions was 0.46 (95% CI 0.13‐0.79; *P*=.03; df=19), indicating significant improvement in the readability of the guidelines following changes to the writing guide in 2020. However, the mean difference in SMOG grade between the LLM-revised and manually revised versions was 0.14 (95% CI −0.18 to 0.45; *P*>.99; df=19), suggesting no difference in the readability of these 2 versions. In contrast, repeated measures ANOVA did not identify differences between the Flesch-Kincaid reading grades of the original, LLM-revised, and manually revised versions (*P*=.91; df=59).

**Table 3.** Summary readability metrics for 3 different versions of injectable medicines guides from practice for 20 intravenous drugs.

	SMOG^a^ grade, mean (SD)	Flesch-Kincaid grade, mean (SD)
Original version	12.3 (0.2)	12.1 (0.4)
LLM^b^-revised version	12.0 (0.2)	12.0 (0.4)
Manually revised version	11.8 (0.1)	11.9 (0.3)

a SMOG: Simple Measure of Gobbledygook.

b LLM: large language model.

The 3 pharmacists’ ratings of the relative readability of the 20 guidelines from practice before and after LLM revision showed a high degree of interrater reliability, with Gwet’s ACs of 0.75 (overall guideline) and 0.85 (subsections). Table 4 presents the pharmacists’ ratings of the overall understandability of the guidelines, which favored the LLM-revised versions being easier to understand (26/60 ratings, 43.3%) compared with the original versions (11/60 ratings, 18.3%). Figure S1 (multimedia supplement) summarizes the pharmacists’ ratings of the relative readability of each subsection. Of 459 individual ratings, 56 favored the original version and 125 favored the LLM-revised version. For 6 of the 9 subsections and 17 of the 20 drugs, there were more ratings that favored the LLM-revised version than the original version. The pharmacists’ free text comments on relative readability frequently mentioned more concise language and a clearer layout for the LLM-revised versions resulting especially from the use of bullet points. However, they also noted that this was sometimes achieved through the omission of information.

**Table 4.** The 3 pharmacists’ ratings of the overall relative understandability of the 20 original and large language model (LLM)–revised guidelines.

	Number of ratings, n (%)^a^
2019 version is much easier to understand	1 (1.7)
2019 version is slightly easier to understand	10 (16.7)
Both versions are equally easy to understand	23 (38.3)
LLM-revised version is slightly easier to understand	25 (41.7)
LLM-revised version is much easier to understand	1 (1.7)

a Three pharmacist reviewers each independently rated 20 guidelines, giving a total of 60 ratings.

### Readability of the User- Tested Guidelines

Table 5 presents the readability metrics for 3 different versions of injectable medicines guides for aminophylline and voriconazole that underwent user testing and subsequent revision in a previous study [9]. For aminophylline, LLM revision did not decrease the reading grade, but following user testing and revision, both readability metrics fell. For voriconazole, LLM revision decreased both readability grades, but user testing and subsequent revision decreased them even further.

**Table 5.** Guideline readability metrics for three different versions of injectable medicines guides for aminophylline and voriconazole that underwent user testing and subsequent revision in a previous study [9].

	Original version	LLM^a^-revised version	User-tested version
SMOG^b^ grade			
Aminophylline	11.7	11.8	11.0
Voriconazole	11.9	10.9	10.6
Flesch-Kincaid grade			
Aminophylline	9.7	9.8	8.3
Voriconazole	10.5	10.3	7.9

a LLM: large language model.

b SMOG: Simple Measure of G﻿obbledygook.

## Discussion

### Principal Findings

The GPT-4–based pipeline did not introduce clinically relevant omissions, additions, or changes of meaning to≥80% of individual subsections within the 20 guidelines from practice. However, content was entirely similar in only a few individual guidelines or subsection types, and differences (especially omissions) were concentrated in certain drugs or subsections in an unpredictable way. It is noteworthy that in the cases of furosemide, phenytoin, and vancomycin, GPT-4 did not maintain a BERT score above 0.9.

SMOG grades suggested that when following similar instructions or prompts, the LLM-based pipeline was as effective as manual editing in producing a small improvement in readability, and this was supported by the expert reviewers’ ratings of understandability. In contrast, the Flesch-Kincaid reading grade did not find any change in readability. This may be because the Flesch-Kincaid reading grade reflects a lower standard of comprehension than the SMOG grade [25] but reinforces the marginal nature of the readability improvement. In addition, the GPT-4 pipeline did not produce improvements in either readability metric as large as those resulting from user testing and subsequent document revision.

### Comparison With Prior Work

These results are aligned with those of studies that have applied LLMs to improve the readability of patient information, which have demonstrated improvements in readability metrics [13-18] that are often associated with the omission of information [13,15,19]. This association between readability and omission of content is unsurprising, as the removal of complex concepts is likely to make a text easier to understand. However, in a safety-critical context, such as health care, the omission of essential information must be avoided, and evidence to date suggests that LLMs are currently unable to achieve this reliably. In contrast, an iterative process of user testing and subsequent document revision has a lower risk of introducing unintended content changes. In addition, while this study has demonstrated that user testing can improve the readability metrics of clinical guidelines, it has also been shown to have beneficial effects on more important user- or patient-centered outcomes, such as reducing difficulties finding and understanding information [8,9,28], and subsequently preventing guideline-related medication errors [11].

Therefore, user testing remains the guideline improvement technique with the strongest evidence base. However, the findings of this study suggest that specifically designed LLM-based pipelines with appropriate safeguards do have potential to augment (but not replace) a manual user testing process, by providing suggestions for potential improvements during the document revision process. These safeguards should be included as explicit clinician-guided, hard-coded programmatic feature checks (eg, verifying that all medications are included and in the correct order) rather than as an instruction to the LLM, which may be ignored. This approach has the potential to partially address the limited availability of knowledge and skills in information writing and design within health care organizations.

### Strengths and Limitations

This study has several strengths, including the use of specific prompts based on previous research and the use of both manually revised and user-tested monographs as comparators. In addition, it evaluated both the content and readability of the guidelines using a combination of calculated metrics and human evaluation, an approach recently recommended for studies in this field [29]. However, there are also a number of limitations. First, user- and patient-centered outcomes might have been evaluated (eg, the ability of end users to find and understand information in the guidelines; the frequency of medication errors when using revised guidelines), so it is not clear whether the observed small improvements in readability would result in improved patient care. This could be addressed by future randomized studies comparing original and LLM-revised guidelines in actual or simulated patient care. Second, this study used a general LLM rather than a bespoke model trained with health care–specific information [29], which might have resulted in greater improvements in readability and/or fewer content changes. Such models should be used in future studies. Third, while the results were replicable among the 22 drug guides tested in this study, only one type of guideline relating to intravenous drug administration was used. Therefore, results may not be replicable to other types of guideline, especially as the prompting strategy was based on user testing of these specific guidelines. Other types of guidelines and more general prompting strategies should be investigated in future studies. Fourth, the inclusion of a prompt based on semantic similarity did not provide a reliable safeguard against content changes, as the BERT score for 3 guidelines was <0.9 (Table 1). Future studies should implement semantic similarity thresholds as programmatic post hoc checks (compute the BERT score externally and rerun or reject LLM outputs) and investigate more extensive use of clinician-guided hard-coded programmatic feature checks (eg, verifying that all medications are included and in the correct order). Fifth, the prompting strategy was developed by only a small team, so it is more likely to have been affected by participant subjectivity. This could be addressed by developing prompts among a larger team with a greater range of expertise. Sixth, instruction 9 in the GPT-4 prompts (“remove information about the size of the bags”) was based on a misinterpretation of previous user testing–derived recommendations. Six of 12 content review panel comments describing omissions from the “Dilution” subsection related to the clinically relevant omission of infusion bag volume, suggesting this error contributed to some of the unintended omissions identified in this study.

### Implications for Practice and Future Research

The present findings suggest that guideline authors should not use current LLMs to modify clinical guidelines without carefully checking the revised text for unintended omissions, additions, or changes of meaning. Similarly, clinicians should not use current LLMs to answer clinical questions without carefully checking the results against reliable sources of information. However, LLMs do have the potential to augment manual user testing and reduce the barriers to the wider use of this approach to improve the safety of the thousands of local clinical guidelines currently in use. Further work is required to embed LLM functionality into guideline authoring systems and subsequently demonstrate the safety and effectiveness of this approach.

### Conclusions

LLMs are able to revise the contents of clinical guidelines to produce small improvements in readability, but with unintended omissions, additions, or changes of meaning to a minority of the information. Small improvements in readability were evident using the SMOG grade and expert review, but not with the Flesch-Kincaid reading grade. It is not known whether these readability improvements would lead to better understanding of, or adherence to, clinical guidelines when used in practice. Omissions of information accounted for the majority of unintended changes to the information contained within the guidelines. Guideline-related medication errors continue to occur but may be prevented by the combination of human expertise, user feedback, and appropriate technologies.

## Supplementary material

10.2196/81915 Multimedia Appendix 1 Large language model prompts and additional data.

## Abbreviations

BERT: bidirectional encoder representations from transformers
IMG: Injectable Medicines Guide
LLM: large language model
SMOG: Simple Measure of Gobbledygook
TRIPOD-LLM: Transparent Reporting of a Multivariable Model for Individual Prognosis or Diagnosis - Large Language Model
