# Supplementary material for: Improving the Understandability of Clinical Guidelines: Development and Evaluation of a GPT-4–Based Pipeline
Source: J Med Internet Res. 2026 Feb 23;28:e81915. doi: 10.2196/81915 (PMC12928683; doi:10.2196/81915)
Supplement: Multimedia Appendix 1 [file jmir-v28-e81915-s001.pdf]

Large language models to improve the understandability of clinical guidelines: an evaluation of readability improvements and unintended content changes produced by GPT-4

Online Supplement

Appendix 1: the final GPT-4 prompts used for both comparisons. These were a product of iterative development, incorporating insights from our explorations while omitting some instructions originally intended for human authors.

Note: Instruction 9 was based on a misinterpretation of previous user-testing derived recommendations and contributed to some of the unintended omissions identified in this study. It should not be included in any future use of these prompts.

“You are a language model tasked with editing a given piece of text to improve clarity and readability. Please follow the specific set of instructions listed below, one by one, to perform the necessary edits. After each edit, please verify the semantic accuracy of the changes before proceeding to the next instruction, if the semantic accuracy is below 0.9 then keep re-iterating until it passes this threshold.

1. If the text is empty:
   - Write "No assessment is given as this requires expert knowledge."
2. If the text only contains the name of the medicine:
   - leave this text unchanged.
3. If the text contains parentheses:
   - Replace parentheses with a dash if it seems appropriate. For example, “Fever and headache (if severe, consider stopping treatment)” becomes “Fever and headache – if severe, consider stopping treatment.”
4. Make sure the first letter of each sentence is capitalised.
5. Change all passive voice sentences to active voice.
6. Combine semantically similar phrases. For example, if the text contains "Do not shake" and "gently swirl," replace it with "gentle swirl."
7. Simplify the sentences to make them easier to read, focus on clarity, however do not change the semantic meaning.

8. If the text contains measurements:

   - Remove the word "to" from mentions of acceptable ranges of measurements. For example, "0.6 mg to 6 mg" should be converted to "0.6-6 mg."

   - Ensure there is a space between the number and the unit. For example "70mg" becomes "70 mg".

9. If the text mentions bags and the size of the bags:

   - Remove information about the size of the bags. For example, if a sentence is "If zyntraxin dose 100-600 mg: add to 30 mL bag" then remove "30 mL" from the sentence, which is the size of the bag.

10. Remove instances of numbers inside parentheses, such as "(1)," that are usually at the end of sentences in bullet points. For example, "Vials containing 100 mg zyntraxin powder for reconstitution. (1) (5)." Remove the "(1) (5)" at the end of the sentence.

11. If the text is about dilution:

   - Explicitly mention the medication names used in each dilution.

12. Check and correct spelling errors, should be in UK English.

13. Do not lose bold markdown.

14. Improve the grammar of the text.

Please proceed to edit the given text below using the provided instructions. Once you have made the necessary improvements, present only the revised version of the text.”

Table S1: Guideline readability metrics for three different versions of Injectable Medicines Guides from practice for 20 intravenous drugs.

	SMOG grade			Flesch-Kincaid grade		
	Original version	LLM-revised version	Manually-revised version	Original version	LLM-revised version	Manually-revised version
Amiodarone	12.7	11.8	11.9	11.7	11.0	10.8
Amoxicillin	11.8	11.5	11.4	10.6	11.0	12.7
Ceftriaxone	12.5	12.1	11.4	10.6	10.1	10.5
Cyclizine	12.7	12.3	12.3	12.5	13.2	12.1
Fentanyl	13.3	12.9	11.7	15.8	15.4	12.5
Flucloxacillin	11.6	11.7	11.7	11.6	11.7	12.9
Furosemide	12.1	13.1	12.5	10.4	12.5	13.0
Gentamicin	13.2	13.1	12.0	14.6	15.6	12.7
Levetiracetam	12.4	11.5	11.8	15.5	14.8	13.8
Magnesium sulfate	12.7	12.3	11.6	12.5	11.1	10.9
Meropenem	12.6	12.5	11.7	11.5	11.4	10.7
Metronidazole	11.2	10.5	11.9	11.5	10.6	13.9
Noradrenaline	13.2	11.9	12.4	13.8	12.8	11.9
Omeprazole	12.2	11.8	11.8	11.6	11.4	11.0
Paracetamol	11.5	11.7	12.4	12.0	12.0	12.2
Phenytoin	13.0	12.7	12.5	11.4	10.8	11.4
Piperacillin and tazobactam	12.6	12.6	12.3	12.3	12.5	13.4
Propofol	12.3	11.8	11.1	12.2	12.9	11.5
Teicoplanin	11.8	11.1	11.0	11.4	10.7	10.7
Vancomycin	10.2	10.2	11.0	7.8	8.0	10.1
Mean (standard deviation)	12.3 (0.2)	12.0 (0.2)	11.8 (0.1)	12.1 (0.4)	12.0 (0.4)	11.9 (0.3)

Figure S1: Heatmap showing number of pharmacists who rated the original or LLM-revised guideline version as 'slightly easier' or 'much easier' to understand for each sub-section. Cells shaded green indicate more ratings favoured the LLM-revised version, and cells shaded red indicate more ratings favoured the original version. Ratings of 'both versions are equally easy to understand' are not shown. Blank sub-sections were not present in the relevant guideline.

	Method		Recon.		Dilution		Expiry		Flush		ADRs		Extrav.		Other		Compat.		Total	
	Original	LLM	Original	LLM	Original	LLM	Original	LLM	Original	LLM	Original	LLM	Original	LLM	Original	LLM	Original	LLM	Original	LLM
Amiodarone	1	2			3	0	0	0	0	0	0	2	0	0	0	0	3	0	7	4
Amoxicillin	2	1	0	1	0	2			0	0	2	1	1	0	0	3	0	2	5	10
Ceftriaxone	1	2	0	3	0	1			0	1	0	3	0	2	0	2	0	3	1	17
Cyclizine	0	2			1	1			0	0	0	3	2	0	0	0	0	1	3	7
Fentanyl	0	2			2	0	1	0	0	0	0	2	0	0	0	2	1	2	4	8
Flucloxacillin	0	1	0	0	1	1			0	0	0	1	0	0	0	1	0	3	1	7
Furosemide	1	2			0	2	0	0	0	0	2	1	3	0	1	1	0	3	7	9
Gentamicin	1	1			0	1			1	2	1	2	1	0	0	2	0	2	4	10
Levetiracetam	1	0			0	0			0	0	0	2	0	0	0	1	0	2	1	5
Magnesium sulfate	2	1			0	1	0	1	0	0	0	2	0	0	0	0	0	3	2	8
Meropenem	0	1	1	1	1	1			0	0	1	1	0	0	0	1	0	2	3	7
Metronidazole	0	2							0	0	0	0	0	0	0	0	1	0	1	2
Noradrenaline	0	1			0	1	0	0	0	0	0	2	0	0	0	0	0	1	0	5
Omeprazole	0	1	0	0	2	1	0	0	0	0	0	1	0	0	0	0	0	1	2	4
Paracetamol	0	1							0	0	0	0	0	0	0	0	0	0	0	1
Phenytoin	0	2			0	1			0	1	1	2			2	0	0	1	3	7
Piperacillin and tazobactam	0	0	0	0	0	0			0	0	0	2	0	0	0	0	0	1	0	3
Propofol	1	1			0	0	0	0	0	0	0	2	0	0	0	1	1	1	2	5
Teicoplanin	0	0	2	0	1	0	0	0	0	0	0	1	0	0	1	0	0	0	4	1
Vancomycin	0	1	2	0	0	1	0	0	0	0	0	1	2	0	2	0	0	2	6	5
**Total**	10	24	5	5	11	14	1	1	1	4	7	31	9	2	6	14	6	30		

Method = Method of Administration; Recon. = Reconstitution; Expiry = Expiry Time; Flush = Flushing; AEs = Adverse Effects; Extrav. = Extravasation; Other = Other Comments; Compat. = Compatibility.
